# Metabolic profiling reveals circulating biomarkers associated with incident and prevalent Parkinson’s disease

**DOI:** 10.1038/s41531-024-00713-2

**Published:** 2024-07-09

**Authors:** Wenyi Hu, Wei Wang, Huan Liao, Gabriella Bulloch, Xiayin Zhang, Xianwen Shang, Yu Huang, Yijun Hu, Honghua Yu, Xiaohong Yang, Mingguang He, Zhuoting Zhu

**Affiliations:** 1 Department of Ophthalmology, Guangdong Provincial People’s Hospital (Guangdong Academy of Medical Sciences), Southern Medical University, Guangzhou, China; 2 Centre for Eye Research Australia; Ophthalmology, University of Melbourne, Melbourne, VIC Australia; 3 Department of Surgery (Ophthalmology), The University of Melbourne, Melbourne, VIC Australia; 4 State Key Laboratory of Ophthalmology, Zhongshan Ophthalmic Center, Sun Yat-sen University, Guangzhou, China; 5 Neural Regeneration Group, Institute of Reconstructive Neurobiology, University of Bonn, Bonn, Germany; 6 School of Optometry, The Hong Kong Polytechnic University, Hong Kong SAR, China; 7 Research Centre for SHARP Vision, The Hong Kong Polytechnic University, Kowloon, Hong Kong SAR, China

**Keywords:** Predictive markers, Parkinson's disease

## Abstract

The metabolic profile predating the onset of Parkinson’s disease (PD) remains unclear. We aim to investigate the metabolites associated with incident and prevalent PD and their predictive values in the UK Biobank participants with metabolomics and genetic data at the baseline. A panel of 249 metabolites was quantified using a nuclear magnetic resonance analytical platform. PD was ascertained by self-reported history, hospital admission records and death registers. Cox proportional hazard models and logistic regression models were used to investigate the associations between metabolites and incident and prevalent PD, respectively. Area under receiver operating characteristics curves (AUC) were used to estimate the predictive values of models for future PD. Among 109,790 participants without PD at the baseline, 639 (0.58%) individuals developed PD after one year from the baseline during a median follow-up period of 12.2 years. Sixty-eight metabolites were associated with incident PD at nominal significance (*P* < 0.05), spanning lipids, lipid constituent of lipoprotein subclasses and ratios of lipid constituents. After multiple testing corrections (*P* < 9$\times$10^−4^), polyunsaturated fatty acids (PUFA) and omega-6 fatty acids remained significantly associated with incident PD, and PUFA was shared by incident and prevalent PD. Additionally, 14 metabolites were exclusively associated with prevalent PD, including amino acids, fatty acids, several lipoprotein subclasses and ratios of lipids. Adding these metabolites to the conventional risk factors yielded a comparable predictive performance to the risk-factor-based model (AUC = 0.766 vs AUC = 0.768, *P* = 0.145). Our findings suggested metabolic profiles provided additional knowledge to understand different pathways related to PD before and after its onset.

## Introduction

Parkinson’s disease (PD) is a multifactorial complex disorder featuring dopaminergic neuron loss and the pathological hallmarks of α-synuclein and Lewy bodies^1^. Although highly efficacious symptomatic therapeutics are available, curative therapies remain scarce^2^. This can be explained by the unclear pathogenesis of neurodegeneration and its insidious onset of 5–6 years predating the onset of typical symptoms^3^. Previous research suggested that mitochondrial dysfunction, defective protein degradation, and oxidative stress are considered important prodromal molecular pathways involved in PD pathogenesis^4^. Nevertheless, the molecular pathways underlying the pathogenesis of PD remain incompletely understood. Therefore, disentangling additional pathways related to the future development of PD is needed for a more comprehensive understanding of the aetiology of PD and helps to reveal potential targets for intervention.

Metabolic profiling is an emerging area of research. Unlike conventional approaches of detecting biomarkers, metabolomics provides a comprehensive roadmap of biological processes in real-time and can reflect the integrated effects of genetics, lifestyle, and environmental factors^5^. This lends itself to the idea that it may unveil novel pathways for multifactorial diseases, such as PD^6^. And the value of metabolite biomarkers to predict health outcomes has been revealed by several previous studies^7,8^.

A series of studies have characterised the metabolomics profile in individuals with PD^9–15^, but distinct analytical techniques used, metabolite panels quantified, and varied statistical analyses that were performed in these studies resulted in inconclusive findings^16^. However, there are a limited number of studies that investigated the metabolomics in individuals predating the onset of PD, where only a few metabolites were found to have a robust association with PD^17^. There is also a lack of evidence on comparing the metabolic profiles between prevalent PD and incident PD, which potentially informs the changes of metabolomics across different stages of PD. Moreover, whether metabolite profiles predating the onset of PD have predictive capabilities of developing future PD remains unclear.

The UK Biobank study is a large prospective study with comprehensive health-related information and has quantified the metabolomic profiles of more than 110,000 participants^18^. With these resources, the current study aims to investigate the metabolite profile alterations associated with incident PD in the large-scale sample derived from the UK Biobank Study and whether metabolites can provide added value for the prediction of future PD risk.

## Results

### Study population

A total of 109,991 participants were included in the present study with a mean (standard deviation [SD]) age of 56.5 (8.10) years, and 53.8% were females. Among the 109,790 participants without PD at baseline, after the median (range and interquartile range [IQR]) follow-up of 12.2 (range: 0.01–14.0, IQR: 11.5–12.9) years, 644 (0.59%) cases of incident PD were identified. Baseline characteristics of the participants stratified by incident PD are shown in Table 1. Participants who developed PD were more likely to be older, male, with higher blood pressure, have a history of diabetes, hyperlipidemia and stroke, take psychotropic medications, and be APOE and GBA variants carriers. The details of the baseline characteristics of participants stratified by prevalent PD at baseline were described in Supplementary Table 2.

**Table 1:** Baseline characteristics of participants stratified by incident PD

Baseline characteristics	Total	Non-PD	PD	*P* value*
*N*	109,790	109,146	644	
Age, mean(SD), years	56.5 (8.10)	56.5 (8.10)	62.7 (5.57)	**<0.001**
Sex, *N* (%)				**<0.001**
Female	59,073 (53.8)	58,838 (53.9)	235 (36.5)	
Male	50,717 (46.2)	50,308 (46.1)	409 (63.5)	
Smoking status, *N* (%)				0.156
Never	59,704 (54.7)	59,372 (54.7)	332 (51.9)	
Prior/current	49,524 (45.3)	49,216 (45.3)	308 (48.1)	
BMI, mean (SD)	27.4 (4.77)	27.4 (4.77)	27.7 (4.50)	0.182
Systolic blood pressure, mean(SD), mmHg	137.7 (18.5)	137.7 (18.5)	141.2 (18.5)	**<0.001**
Currently on anti-hypertensive treatments, *N* (%)				0.289
No	99,368 (90.5)	98,793 (90.5)	575 (89.3)	
Yes	10,422 (9.49)	10,353 (9.49)	69 (10.7)	
History of diabetes mellitus, *N* (%)				**<0.001**
No	103,134 (93.9)	102,574 (94.0)	560 (87.0)	
Yes	6656 (6.06)	6572 (6.02)	84 (13.0)	
History of hyperlipidemia, *N* (%)				**0.002**
No	58,947 (53.7)	58,640 (53.7)	307 (47.7)	
Yes	50,843 (46.3)	50,506 (46.3)	337 (52.3)	
Prior stroke, *N* (%)				**<0.001**
No	108,263 (98.6)	107,643 (98.6)	620 (96.3)	
Yes	1527 (1.39)	1503 (1.38)	24 (3.73)	
Use of psychotropic medications, *N* (%)				**0.008**
No	100,974 (92.0)	100,400 (92.0)	574 (89.1)	
Yes	8816 (8.03)	8746 (8.01)	70 (10.9)	
Presence of APOE allelic variant(s), *N* (%)				**0.004**
No	83,184 (75.8)	82,727 (75.8)	457 (71.0)	
Yes	26,606 (24.2)	26,419 (24.2)	187 (29.0)	
Presence of GBA allelic variant(s), *N*(%)				**<0.001**
No	104,519 (95.2)	103,930 (95.2)	589 (91.5)	
Yes	5217 (4.80)	5216 (4.78)	55 (8.54)	

*A P value less than 0.05 indicated statistical significance and was bold.

### Circulating metabolites associated with incident PD

**Fig. 1:**
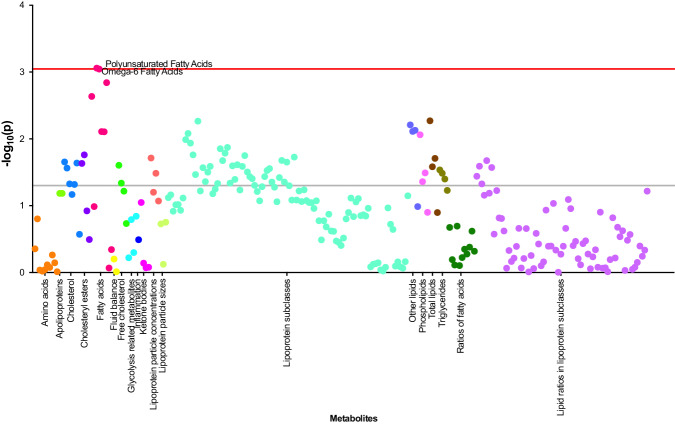
Manhattan plot of all 249 metabolites investigated for incident PD. All 249 metabolites were categorised by 19 sub-groups. Metabolites above the grey line indicate metabolites associated with PD at nominal significance (*P* < 0.05) and those annotated above the red line indicate a significant association after multiple testing corrections (*P* < 9$\times$10^−4^). *P* values for the association between each metabolite and PD risk were derived from Wald tests.

**Fig. 2:**
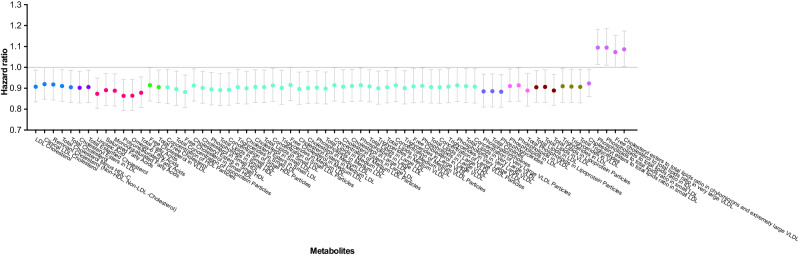
Forest plot of 68 metabolites associated with PD at nominal significance. The estimated scales and directions of the associations (expressed as hazard ratios and 95% confidence intervals) between 68 metabolites and incident PD.

Given the chronic onset of PD, we excluded the participants who developed PD within one year from baseline when exploring the association between plasma metabolites and incident PD. The results of the metabolic associations for the remaining 639 incident PD cases are shown in Figs. 1, 2 and Supplementary Table 4. After adjusting for all covariates, 68 metabolites among the 249 measured metabolites were associated with incident PD at nominal significance, spanning lipid subgroups (cholesterol, cholesterol esters, fatty acids [FA], free cholesterol, phospholipids, triglycerides, and other lipids), lipoprotein particle concentrations, total lipids, lipid constituent of lipoprotein subclasses and ratios of lipid constituents. All metabolites were inversely associated with incident PD except for four metabolites in the subgroup of lipid ratios in lipoprotein subclasses: cholesteryl esters to total lipids ratio in chylomicrons and extremely large very-low-density lipoprotein (VLDL), phospholipids to total lipids ratio in intermediate-density lipoprotein (IDL) and in small low-density lipoprotein (LDL), and free cholesterol to total lipids ratio in very large VLDL (Fig. 2).

After correcting for multiple comparisons, two metabolites including omega-6 FA (HR = 0.86, 95% CI: 0.79–0.94, *P* = 8.71$\times$10^−4^) and polyunsaturated FA (PUFA) (HR = 0.86, 95% CI: 0.79–0.94, *P* = 8.98$\times$10^−4^) remained to be significantly associated with incident PD (Figs. 1, 2 and Supplementary Table 4).

After further adjusting the frailty index in the model, the results were similar to the main analysis (Supplementary Table 3). 92 metabolites were identified to be associated with incident PD, with omega-6 FA and PUFA remaining significant associations with incident PD.

### Circulating metabolites comparison between prevalent and incident PD

Participants who developed PD within one year from the baseline were considered to be in the prevalent PD group. A total of 133 metabolites were associated with prevalent PD at nominal significance and 15 metabolites were associated with prevalent PD after multiple testing corrections (Supplementary Table 4). At nominal significance, 56 metabolites were shared by both prevalent and incident PD, falling in categories of lipoprotein subclasses, FA, free cholesterol, lipoprotein particle concentrations, triglycerides, cholesterol, cholesterol esters, choline, phospholipids, and total lipids. After multiple testing corrections, prevalent and incident PD shared one overlapping metabolite, PUFA. Omega-6 FA was exclusively associated with incident PD, and 14 metabolites were exclusively associated with prevalent PD, including amino acids (tyrosine and valine), FAs (omega-3 FA, DHA, and total FA), different lipoprotein subclasses and ratios of lipids.

### Predictive value of added metabolites in 10-year PD risk

Supplementary Fig. 1 demonstrated the receiver operating characteristic (ROC) curves of conventional risk factor-based model, the metabolite-based model and the combined model for prediction of PD risk in 10 years. The AUC for conventional risk factors-based model was 0.766 (95% CI: 0.746–0.787). The model comprising omega-6 FA and PUFA achieved an AUC of 0.580 (95% CI: 0.554–0.607). The AUC for adding omega-6 FA and PUFA to the risk-factor-based model was 0.768 (95% CI: 0.748–0.788). Despite the slight increase in the predictive value, performance of the combined model was comparable to the risk-factor-based model (*P* = 0.145).

## Discussion

Our study identified 68 metabolites associated with incident PD including lipids, all sizes of lipoprotein subclasses, and their concentrations and ratios of lipid constituents. Two metabolites including PUFA and omega-6 FA remain significant inverse associations with PD after correction for multiple testing and PUFA was the metabolite shared by prevalent and incident PD. Many metabolites were supported by previous studies examining the molecular signatures of PD, including lipoproteins and lipid constituents. The present study provided additional evidence on all sizes of lipoprotein subclasses (chylomicron and extremely large VLDL, VLDL, LDL, and HDL), their lipid constituents and ratios of components. These findings help to provide additional evidence to understand different pathways related to the development of PD.

Fatty acid metabolism was one of the most important pathways found in the analyses, with several most robust biomarkers of PD falling into this category. Total FA, saturated FA, monounsaturated fatty acids, PUFA, omega-3 FA and omega-6 FA-two classes of PUFA, and linoleic acid-a short-chain omega-6 FA were found to be protective against PD in our study. Previous prospective longitudinal studies and clinical trials have provided supportive evidence of the inverse association between the intake of fatty acids and the risk of PD^19–22^. The neuroprotective properties of unsaturated FA could be attributed to their proven properties of inflammation resolution, immune modulation, and oxidative stress alleviation^23–26^. Specifically, PUFA derivatives had the ability to modulate dopaminergic activity in the basal ganglia^27^. In vitro, studies have found that omega-6 FA (linoleic acid and arachidonic acid) could resurrect the viability of MPP-induced Parkinsonism cell models^28^ which could explain our findings on protective benefits of omega-6 FA. However, our findings are the first to suggest an association of saturated FA with incident PD. Previous studies on dietary intake found no association between saturated FA and incident PD^20,22^ or even adverse effects of saturated FA in animal experiments^29^. Considering the differences between simple dietary intake and the complex metabolism procedure and between humans and rats, these findings warrant further investigations in cohort studies.

Lipoproteins of various particle sizes and subclasses (total cholesterol, LDL-cholesterol, HDL-cholesterol, VLDL-cholesterol and chylomicrons) were inversely associated with PD in the present study. These findings were supported by previous studies^30–34^, although inconsistent results were found in some research^31,35,36^. The potential mechanism underlying the association could be the critical neuroprotective role of lipids in repairing or alleviating PD pathology in the central nervous system (CNS)^20,35^. Higher levels of cholesterol could be recruited to sustain synaptogenesis^37^ or sequester ferrous iron to prevent iron-induced oxidative stress^38^. Alternatively, given that the brain can synthesise cholesterol de novo^39^, the involvement of lipids in PD pathogenesis might start in the periphery, such as the gut^31^. For example, α-synuclein (α-syn) accumulation found in the enteric nervous system may predate CNS pathology and its presence may be an indicator of early PD pathogenesis^40^. Indeed, gastrointestinal symptoms are common in PD patients, and malnutrition is among the manifestations, which provides the rationale for the lower lipid levels found in these patients^41^. Research on VLDL-cholesterol and chylomicrons is relatively limited. Only one cross-sectional study found lower serum VLDL-cholesterol was associated with PD^42^, partially supporting our findings of VLDL as protective. As these are triglyceride-rich lipoproteins, the inverse association of chylomicrons with PD might be attributed to the makeup of the molecule being largely triglyceride and cholesterol.

Triglycerides and cholines were also found to be negatively associated with incident PD. A growing number of studies and meta-analyses have suggested the role of triglycerides in PD is protective^35,36,43^. Moreover, Laguna et al. found decreased triglycerides in a specific lipoprotein subclass (LDL) during the conversion from prodromal phase to Lewy body dementia (DLB) which shared similar hallmark pathology with PD^44^. Cholines are an essential nutrient critical for brain development and basic biological processes in cells such as the synthesis of plasma membrane lipids^45–47^. Decreased levels of specific cholines such as phosphatidylcholines and sphingomyelins have been found in the various brain structures of PD patients^48–50^.

Interestingly, among 68 metabolites associated with PD, only four metabolites were positively associated with incident PD, which were all ratios of component lipids in different sizes of lipoprotein particles. It could be postulated that, although these lipids were considered protective when examined separately, their interplay and balance could hold meaning to biological processes that are yet to be understood. The value of lipoproteins ratios is an emerging area of research in cardiovascular diseases, and some ratios may even be better indicators of risk prediction than lipoproteins in isolation^51–54^. There are limited studies on these biomarkers in neurodegenerative diseases, so the implications of these biomarkers require further scrutiny.

Although we identified metabolites that were significantly associated with PD, the improvement in the predictive value by adding them to the conventional risk-factor-based model was subtle. This may potentially be explained by the involvement of PD risk factors in the model, such as diabetes, hypertension and hyperlipidemia per se already demonstrated some changes in the metabolic profile, which masked the added value of the selected metabolites. Nevertheless, our study provided additional insight into the underlying mechanism of PD through the aspects of metabolites including the detailed breakdown of the lipid constituents and ratios of lipids in different sizes of lipoproteins which were rarely studied previously. We also examined the metabolic profiles of prevalent and incident PD and found the exclusive and overlapping metabolites between different disease states, which potentially provides clues for future studies to corroborate the dynamics of molecular pathways.

PD is increasingly considered as a complex heterogeneous disorder that has been classified into different subtypes in terms of clinical, hereditary, imaging and pathological features^55^. Whether different molecular pathways are involved in different subtypes remains to be elucidated^55^. Further studies are warranted to investigate metabolomics in the context of different subtypes of PD to increase the specificity and explore the potential additional value of this tool. Moreover, the protocol of blood sampling in the UK Biobank prevented us from understanding the potential fluctuations of the metabolite levels throughout the day, which needs to be considered when interpreting the results. Further studies should streamline the protocol of sample collection with the aim to delve deeper into the influences of timing on the metabolic profile of PD. In addition, although we compared the metabolic profiles between prevalent and incident PD, future studies are needed to investigate the longitudinal changes of the metabolomic profiles of participants from prodromal to clinical onset of PD. This will help to reveal the trajectory of the changes in the metabolic profile and therefore, enable the selection of target biomarkers for early intervention or evaluation of treatment response.

There are several strengths of our study including a large sample size and long-term follow-up period. In addition, nuclear magnetic resonance (NMR) spectroscopy demonstrates higher reproducibility and quantitative capabilities than mass spectrometry^56^. However, there are several limitations to mention. First, NMR platforms have lower sensitivity and selectivity, making it difficult to detect metabolites at very low concentrations for targeted analysis^56^. Second, despite the longitudinal design of the study, we could infer potential risk factors for incident PD but could not postulate the causality. Therefore, our results warrant further investigation by interventional studies. Thirdly, the UK Biobank study consists of a generally healthy, Caucasian cohort which may make it prone to health selection bias. Despite this, the representativeness of PD within the sample would not affect our associations^57^. Fourthly, although we adopted an algorithm-based strategy for incident PD ascertainment, there is a possibility that incident PD cases were underestimated because of the lag between disease onset and diagnosis. Fifth, we selected the risk factors for PD risk prediction from the previous literature instead of using the Movement Disorder Society (MDS) criteria for prodromal PD, given the difficulty to define all the markers using the UK Biobank data. Moreover, the results were not validated in external datasets, and some metabolomics data were derived from absolute values, calling for future external validation of the metabolic associations and validity of the derived metabolite indices. Lastly, we could not exclude residual confounding.

In the present study, we identified lipids, apolipoproteins, lipoprotein subclasses, and their concentrations and ratios of lipid constituents to be metabolites associated with incident PD. In addition, the metabolic profiles between prevalent and incident PD were different but shared certain common metabolites. These findings suggested that metabolic profiles can provide additional insights to understand the pathogenesis of PD.

## Methods

### Study sample

The study sample was derived from the UK Biobank study, a cohort consisting of more than 500,000 participants aged 40–69 years across the UK^18^. Baseline recruitment was performed from 2006 to 2010 with comprehensive health-related information collected, and additional data were regularly augmented. Repeating visits and online follow-ups were also performed, and health outcomes were tracked longitudinally through electronic health-related records. A detailed protocol of the UK Biobank has been described in a previous study^18^. In the present study, participants with complete data on quantified metabolites and genetic data at baseline (*n* = 109,991) were included. Of these, 201 individuals had a history of PD at baseline, and five participants developed PD within one year from the baseline. Considering the chronic onset of PD, the remaining 109,785 participants were included in the analysis of the metabolic associations with incident PD. In order to compare the metabolic profiles between prevalent and incident PD, a total of 109,991 participants, including 201 participants with diagnosed PD at the baseline and five participants diagnosed with PD within one year from the baseline were included in the association study between metabolites and prevalent PD.

### Metabolites quantification

Details of the NMR platform and experimentation have been described elsewhere^54^. Nightingale Health (Finland) quantified the metabolites of EDTA plasma samples from approximately 120,000 UK Biobank participants. 118,000 samples were collected at baseline of the UK Biobank study, and 5000 were obtained at the repeat assessment. Samples were measured from June 2019 and April 2020. The blood samples were collected from participants in a non-fasting state, but they were advised to have at least four hours of fasting beforehand^58^. A total of 249 metabolites (Supplementary Table 1) were quantified, including lipoprotein lipids of 14 different particle sizes, FA, amino acids, ketone bodies, and glycolysis metabolites.

### Definition of PD

PD was defined by the UK Biobank algorithm using combined sources of self-reported PD through questionnaire and nurse-led interviews, hospital admission records and death registry (see details at http://www.ukbiobank.ac.uk). Vascular parkinsonism and atypical parkinsonism such as multiple system atrophy (MSA) and progressive supranuclear palsy were excluded. In hospital admission records and death registries, PD was ascertained by the International Classification of Diseases (ICD-9) codes 332.0 and ICD-10 codes G20. Participants with PD at baseline were ascertained by self-report of PD diagnosis, and hospital admission records. Incident PD was ascertained by hospital admission records and death registers after the baseline assessment. Follow-up periods were from baseline to the first occurrence of PD, death date, or the last follow-up date, whichever is the earliest.

### Covariates

By reviewing previous studies, covariates in the present study included baseline age, sex, smoking status, body mass index (BMI), systolic blood pressure (SBP), treated hypertension, history of diabetes, history of hyperlipidemia, history of stroke and use of psychotropic medications^33,59–64^. Additional covariates included the GBA variants which increase the risk of PD and affect the levels of plasma apolipoproteins^65,66^, and the presence of APOE allelic variants, which interfere with the lipoprotein levels in PD patients^67^. Age, BMI, and SBP were treated as continuous variables, and other variables were categorised as yes or no.

Treated hypertension was defined by participant-reported treatment on anti-hypertensive medications. History of diabetes was defined as with an HbA1c level $\ge$6.5%, with a diagnosis of diabetes, taking anti-diabetic medications, or on insulin treatment. History of hyperlipidemia was defined as having self-reported hyperlipidemia, taking anti-dyslipidemia medications, or a cholesterol level of 6.21 mmol/L and higher. History of stroke was defined by the first occurrence of stoke if preceding the baseline assessment. Psychotropic medications included anti-depressive, anti-migraine, and anxiolytic medications.

### Statistical analyses

For the plasma metabolites, a natural logarithmic transformation was applied to the raw data and *Z* score normalisation was further performed. Descriptive statistics summarised and organised our data. Categorical variables were described as numbers and percentages, and continuous variables were described with means and SDs or medians and IQRs. Comparison of the variables across different groups was carried out using the non-paired *t* tests continuous variables or Chi-square tests for categorical variables. A two-sided *P* value less than 0.05 was considered statistically significant for the above tests.

Cox proportional hazard models were applied to model the association between metabolites and incident PD. Logistic regression models were used to evaluate the associations between metabolic profiles and prevalent PD at baseline. Multivariable-adjusted models were conducted. The confounders adjusted in the main model were age, sex, smoking status, BMI, SBP, treated hypertension, history of diabetes, history of hyperlipidemia, stroke, use of psychotropic medications, presence of APOE allelic variants and GBA variants. We conducted an additional model by further adjusting for frailty index to investigate the association between metabolites and incident PD^68^. The frailty index was defined by a previously published method in the UK Biobank population, combining five aspects: weight loss, feeling of exhaustion, physical inactivity, walking speed and grip strength^69^. Hazard ratio (HR), odds ratios (OR) and 95% confidence intervals estimated the associations for which a *P* value less than 0.05 was considered nominally significant. Considering the potential strong correlation between the metabolites, we performed a principal component analysis (PCA) according to a previously described method^70^ and found that 55 components could account for 99.5% of the total differences. Therefore, we considered a *p* value of less than nine$\times$10^−4^ (0.05/55) as statistically significant. Wald tests were employed to assess the statistical significance of the estimated HRs and ORs for each metabolite.

Three logistic regression models were established to test the predictive values of conventional risk factors and specific metabolites for future PD development in 10 years. Model 1 included traditional risk factors determined by reviewing previous research: baseline age, sex, smoking status, BMI, SBP, treated hypertension, history of diabetes, history of hyperlipidemia, history of stroke, use of psychotropic medications, and the presence of GBA variants^33,59–65^. Notably, these factors are not chosen based on the MDS Research Criteria for Prodromal PD^71^, given the lack of data to define the whole set of risk markers and prodromal markers in the UK Biobank. Model 2 was based on metabolites associated with incident PD after correction for multiple tests. Model 3 combined all variables in Model 1 and Model 2. Area under receiver operating characteristics curves (AUC) were used to estimate the performance of each model. DeLong test was employed to assess the statistical significance of the difference between AUCs.

All statistical analyses were performed using Stata version 13 (StataCorp LLC, College Station, Texas USA).

### Reporting summary

Further information on research design is available in the Nature Research Reporting Summary linked to this article.

### Supplementary information

Supplementary Material

Reporting summary

## Data Availability

The data used in this study is obtained from the UK Biobank. The UK Biobank data is available to all bona fide researchers through the application (https://www.ukbiobank.ac.uk/).

## Code availability

The code employed for data analysis can be obtained by contacting the corresponding author upon request.
