# Supplementary material for: Metabolic profiling reveals circulating biomarkers associated with incident and prevalent Parkinson’s disease
Source: NPJ Parkinsons Dis. 2024 Jul 9;10:130. doi: 10.1038/s41531-024-00713-2 (PMC11233508; doi:10.1038/s41531-024-00713-2)
Supplement: Supplementary file 2 — Reporting summary [file 41531_2024_713_MOESM2_ESM.pdf]

nature portfolio

Corresponding author(s): Dr Zhuoting Zhu

Last updated by author(s): Mar 6, 2024

Reporting Summary

Nature Portfolio wishes to improve the reproducibility of the work that we publish. This form provides structure for consistency and transparency in reporting. For further information on Nature Portfolio policies, see our Editorial Policies and the Editorial Policy Checklist.

Statistics

For all statistical analyses, confirm that the following items are present in the figure legend, table legend, main text, or Methods section.

n/a	Confirmed	
☐	☒	The exact sample size (*n*) for each experimental group/condition, given as a discrete number and unit of measurement
☒	☐	A statement on whether measurements were taken from distinct samples or whether the same sample was measured repeatedly
☐	☒	The statistical test(s) used AND whether they are one- or two-sided<br>*Only common tests should be described solely by name; describe more complex techniques in the Methods section.*
☐	☒	A description of all covariates tested
☐	☒	A description of any assumptions or corrections, such as tests of normality and adjustment for multiple comparisons
☐	☒	A full description of the statistical parameters including central tendency (e.g. means) or other basic estimates (e.g. regression coefficient) AND variation (e.g. standard deviation) or associated estimates of uncertainty (e.g. confidence intervals)
☐	☒	For null hypothesis testing, the test statistic (e.g. *F*, *t*, *r*) with confidence intervals, effect sizes, degrees of freedom and *P* value noted<br>*Give P values as exact values whenever suitable.*
☒	☐	For Bayesian analysis, information on the choice of priors and Markov chain Monte Carlo settings
☒	☐	For hierarchical and complex designs, identification of the appropriate level for tests and full reporting of outcomes
☐	☒	Estimates of effect sizes (e.g. Cohen's *d*, Pearson's *r*), indicating how they were calculated

Our web collection on statistics for biologists contains articles on many of the points above.

Software and code

Policy information about availability of computer code

Data collection	No software was used.
Data analysis	All statistical analyses were performed using Stata version 13 (StataCorp LLC, College Station, Texas USA).

For manuscripts utilizing custom algorithms or software that are central to the research but not yet described in published literature, software must be made available to editors and reviewers. We strongly encourage code deposition in a community repository (e.g. GitHub). See the Nature Portfolio guidelines for submitting code & software for further information.

Data

Policy information about availability of data

All manuscripts must include a data availability statement. This statement should provide the following information, where applicable:

- Accession codes, unique identifiers, or web links for publicly available datasets
- A description of any restrictions on data availability
- For clinical datasets or third party data, please ensure that the statement adheres to our policy

The data used in this study is obtained from the UK Biobank. The UK Biobank data is available to all bona fide researchers through application (https://www.ukbiobank.ac.uk/). The current study was conducted using UK Biobank data under Application ID 94372.

Research involving human participants, their data, or biological material

Policy information about studies with human participants or human data. See also policy information about sex, gender (identity/presentation), and sexual orientation and race, ethnicity and racism.

Reporting on sex and gender	We used the biological term "sex" in this study. Participants of both sexes were included in this study. We described the characteristics of the cohort in terms of sex but no sex-based analysis was performed. We are focusing the association between metabolites and Parkinson's disease regardless of sex.
Reporting on race, ethnicity, or other socially relevant groupings	This study did not included socially relevant groupings. We did not discuss ethnicity performed analysis based on ethnicity. We included the confounding factors that are relevant to Parkinson's disease and plasma metabolite levels.
Population characteristics	Population characteristics were described in this study including baseline age, sex, smoking status, body mass index (BMI), systolic blood pressure (SBP), treated hypertension, history of diabetes, history of hyperlipidemia, history of stroke and use of psychotropic medications. Additional covariates included the GBA variants and APOE allelic variants.
Recruitment	The study sample was derived from the UK biobank study, consisting of more than 500,000 participants aged 40-69 years across the UK. Baseline recruitment was performed through 2006-2010 with comprehensive health-related information collected, and additional data were regularly augmented. Repeating visits and online follow-up were performed, and health outcomes were tracked longitudinally through electronic health-related records (EHR).
Ethics oversight	Ethical Approval<br>The UK Biobank has obtained ethical approvals from the North West Multi-centre Research Ethics Committee (MREC) (11/NW/0382), which covers the UK. The current study was conducted using UK Biobank data under Application ID 94372. All participants provided written informed consent. The study was conducted adhering to the tenets of the declarations of Helsinki.

Note that full information on the approval of the study protocol must also be provided in the manuscript.

Field-specific reporting

Please select the one below that is the best fit for your research. If you are not sure, read the appropriate sections before making your selection.

☒ Life sciences ☐ Behavioural & social sciences ☐ Ecological, evolutionary & environmental sciences

For a reference copy of the document with all sections, see nature.com/documents/nr-reporting-summary-flat.pdf

Life sciences study design

All studies must disclose on these points even when the disclosure is negative.

Sample size	In the present study, participants with complete data on quantified metabolites and genetic data at baseline (n=109,991) were included. Of these, 201 individuals had a history of PD at baseline, and 5 participants developed PD within one year from the baseline.
Data exclusions	Considering the chronic onset of PD, the remaining 109,785 participants were included in the analysis of the metabolic associations with incident PD. In order to compare the metabolic profiles between prevalent and incident PD, a total of 109,991 participants, including 201 participants with diagnosed PD at the baseline and 5 participants diagnosed with PD within one year from the baseline were included in the association study between metabolites and prevalent PD.
Replication	n/a
Randomization	n/a
Blinding	n/a

Reporting for specific materials, systems and methods

We require information from authors about some types of materials, experimental systems and methods used in many studies. Here, indicate whether each material, system or method listed is relevant to your study. If you are not sure if a list item applies to your research, read the appropriate section before selecting a response.

Materials & experimental systems

n/a	Involved in the study
☒	☐ Antibodies
☒	☐ Eukaryotic cell lines
☒	☐ Palaeontology and archaeology
☒	☐ Animals and other organisms
☒	☐ Clinical data
☒	☐ Dual use research of concern
☒	☐ Plants

Methods

n/a	Involved in the study
☒	☐ ChIP-seq
☒	☐ Flow cytometry
☒	☐ MRI-based neuroimaging

Plants

Seed stocks	n/a
Novel plant genotypes	n/a
Authentication	n/a
