# Supplementary material for: Metabolic profiling reveals circulating biomarkers associated with incident and prevalent Parkinson’s disease
Source: NPJ Parkinsons Dis. 2024 Jul 9;10:130. doi: 10.1038/s41531-024-00713-2 (PMC11233508; doi:10.1038/s41531-024-00713-2)
Supplement: Supplementary file 1 — Supplementary Material [file 41531_2024_713_MOESM1_ESM.pdf]

Supplementary Materials

Supplementary Table 1. The names and the group names of the plasma metabolites analysed.

No.	Metabolites	Group
1	Glutamine	Amino acids
2	Alanine	Amino acids
3	Phenylalanine	Amino acids
4	Tyrosine	Amino acids
5	Histidine	Amino acids
6	Glycine	Amino acids
7	Isoleucine	Amino acids
8	Valine	Amino acids
9	Total Concentration of Branched-Chain Amino Acids (Leucine + Isoleucine + Valine)	Amino acids
10	Leucine	Amino acids
11	Apolipoprotein B	Apolipoproteins
12	Apolipoprotein A1	Apolipoproteins
13	Remnant Cholesterol (Non-HDL, Non-LDL -Cholesterol)	Cholesterol
14	Total Cholesterol Minus HDL-C	Cholesterol
15	VLDL Cholesterol	Cholesterol
16	Total Cholesterol	Cholesterol
17	LDL Cholesterol	Cholesterol
18	Clinical LDL Cholesterol	Cholesterol
19	HDL Cholesterol	Cholesterol
20	Cholesteryl Esters in LDL	Cholesteryl esters
21	Total Esterified Cholesterol	Cholesteryl esters
22	Cholesteryl Esters in VLDL	Cholesteryl esters
23	Cholesteryl Esters in HDL	Cholesteryl esters
24	Omega-6 Fatty Acids	Fatty acids
25	Linoleic Acid	Fatty acids
26	Polyunsaturated Fatty Acids	Fatty acids
27	Total Fatty Acids	Fatty acids
28	Monounsaturated Fatty Acids	Fatty acids
29	Saturated Fatty Acids	Fatty acids
30	Omega-3 Fatty Acids	Fatty acids
31	Degree of Unsaturation	Fatty acids
32	Docosahexaenoic Acid	Fatty acids
33	Creatinine	Fluid balance
34	Albumin	Fluid balance
35	Free Cholesterol in VLDL	Free cholesterol
36	Total Free Cholesterol	Free cholesterol
37	Free Cholesterol in LDL	Free cholesterol
38	Free Cholesterol in HDL	Free cholesterol

39	Pyruvate	Glycolysis related metabolites
40	Citrate	Glycolysis related metabolites
41	Lactate	Glycolysis related metabolites
42	Glucose	Glycolysis related metabolites
43	Glycoprotein Acetyls	Inflammation
44	Acetate	Ketone bodies
45	3-Hydroxybutyrate	Ketone bodies
46	Acetoacetate	Ketone bodies
47	Acetone	Ketone bodies
48	Concentration of VLDL Particles	Lipoprotein particle concentrations
49	Concentration of LDL Particles	Lipoprotein particle concentrations
50	Total Concentration of Lipoprotein Particles	Lipoprotein particle concentrations
51	Concentration of HDL Particles	Lipoprotein particle concentrations
52	Average Diameter for VLDL Particles	Lipoprotein particle sizes
53	Average Diameter for LDL Particles	Lipoprotein particle sizes
54	Average Diameter for HDL Particles	Lipoprotein particle sizes
55	Triglycerides in Medium LDL	Lipoprotein subclasses
56	Cholesteryl Esters in Medium LDL	Lipoprotein subclasses
57	Cholesteryl Esters in Small LDL	Lipoprotein subclasses
58	Total Lipids in Medium LDL	Lipoprotein subclasses
59	Triglycerides in Large LDL	Lipoprotein subclasses
60	Total Lipids in Medium VLDL	Lipoprotein subclasses
61	Cholesterol in Medium LDL	Lipoprotein subclasses
62	Concentration of Medium VLDL Particles	Lipoprotein subclasses
63	Total Lipids in Small LDL	Lipoprotein subclasses
64	Total Lipids in Small VLDL	Lipoprotein subclasses
65	Triglycerides in Small LDL	Lipoprotein subclasses
66	Phospholipids in Medium LDL	Lipoprotein subclasses
67	Phospholipids in Medium VLDL	Lipoprotein subclasses
68	Cholesterol in Small LDL	Lipoprotein subclasses
69	Concentration of Small LDL Particles	Lipoprotein subclasses
70	Triglycerides in Medium VLDL	Lipoprotein subclasses
71	Phospholipids in Small VLDL	Lipoprotein subclasses
72	Concentration of Medium LDL Particles	Lipoprotein subclasses
73	Concentration of Small VLDL Particles	Lipoprotein subclasses
74	Free Cholesterol in Large VLDL	Lipoprotein subclasses
75	Triglycerides in IDL	Lipoprotein subclasses
76	Concentration of Large VLDL Particles	Lipoprotein subclasses

77	Cholesterol in Large VLDL	Lipoprotein subclasses
78	Free Cholesterol in Small HDL	Lipoprotein subclasses
79	Total Lipids in Large VLDL	Lipoprotein subclasses
80	Free Cholesterol in Medium VLDL	Lipoprotein subclasses
81	Triglycerides in Very Large HDL	Lipoprotein subclasses
82	Total Lipids in Large LDL	Lipoprotein subclasses
83	Concentration of IDL Particles	Lipoprotein subclasses
84	Cholesteryl Esters in Large VLDL	Lipoprotein subclasses
85	Cholesterol in Very Large VLDL	Lipoprotein subclasses
86	Triglycerides in Small VLDL	Lipoprotein subclasses
87	Phospholipids in Large VLDL	Lipoprotein subclasses
88	Free Cholesterol in Very Large VLDL	Lipoprotein subclasses
89	Concentration of Very Large VLDL Particles	Lipoprotein subclasses
90	Triglycerides in Large VLDL	Lipoprotein subclasses
91	Cholesteryl Esters in Very Large VLDL	Lipoprotein subclasses
92	Concentration of Very Small VLDL Particles	Lipoprotein subclasses
93	Cholesteryl Esters in Large LDL	Lipoprotein subclasses
94	Phospholipids in Large LDL	Lipoprotein subclasses
95	Triglycerides in Very Small VLDL	Lipoprotein subclasses
96	Phospholipids in Small LDL	Lipoprotein subclasses
97	Phospholipids in Very Large VLDL	Lipoprotein subclasses
98	Total Lipids in Very Small VLDL	Lipoprotein subclasses
99	Triglycerides in Small HDL	Lipoprotein subclasses
100	Cholesterol in Small VLDL	Lipoprotein subclasses
101	Free Cholesterol in Small VLDL	Lipoprotein subclasses
102	Total Lipids in Very Large VLDL	Lipoprotein subclasses
103	Total Lipids in IDL	Lipoprotein subclasses
104	Cholesterol in Large LDL	Lipoprotein subclasses
105	Cholesteryl Esters in Small VLDL	Lipoprotein subclasses
106	Free Cholesterol in Very Small VLDL	Lipoprotein subclasses
107	Phospholipids in Very Small VLDL	Lipoprotein subclasses
108	Triglycerides in Very Large VLDL	Lipoprotein subclasses
109	Cholesteryl Esters in IDL	Lipoprotein subclasses
110	Free Cholesterol in Medium LDL	Lipoprotein subclasses
111	Triglycerides in Medium HDL	Lipoprotein subclasses
112	Cholesterol in IDL	Lipoprotein subclasses
113	Phospholipids in IDL	Lipoprotein subclasses
114	Total Lipids in Small HDL	Lipoprotein subclasses
115	Concentration of Large LDL Particles	Lipoprotein subclasses
116	Cholesterol in Medium VLDL	Lipoprotein subclasses
117	Free Cholesterol in Chylomicrons and Extremely Large VLDL	Lipoprotein subclasses
118	Cholesterol in Chylomicrons and Extremely Large VLDL	Lipoprotein subclasses
119	Free Cholesterol in IDL	Lipoprotein subclasses

120	Free Cholesterol in Large LDL	Lipoprotein subclasses
121	Cholesteryl Esters in Chylomicrons and Extremely Large VLDL	Lipoprotein subclasses
122	Phospholipids in Small HDL	Lipoprotein subclasses
123	Concentration of Chylomicrons and Extremely Large VLDL Particles	Lipoprotein subclasses
124	Triglycerides in Large HDL	Lipoprotein subclasses
125	Cholesterol in Very Small VLDL	Lipoprotein subclasses
126	Concentration of Small HDL Particles	Lipoprotein subclasses
127	Phospholipids in Chylomicrons and Extremely Large VLDL	Lipoprotein subclasses
128	Total Lipids in Chylomicrons and Extremely Large VLDL	Lipoprotein subclasses
129	Cholesteryl Esters in Very Small VLDL	Lipoprotein subclasses
130	Triglycerides in Chylomicrons and Extremely Large VLDL	Lipoprotein subclasses
131	Free Cholesterol in Small LDL	Lipoprotein subclasses
132	Cholesteryl Esters in Medium VLDL	Lipoprotein subclasses
133	Cholesterol in Small HDL	Lipoprotein subclasses
134	Cholesteryl Esters in Small HDL	Lipoprotein subclasses
135	Phospholipids in Medium HDL	Lipoprotein subclasses
136	Free Cholesterol in Medium HDL	Lipoprotein subclasses
137	Total Lipids in Medium HDL	Lipoprotein subclasses
138	Concentration of Medium HDL Particles	Lipoprotein subclasses
139	Cholesterol in Medium HDL	Lipoprotein subclasses
140	Concentration of Very Large HDL Particles	Lipoprotein subclasses
141	Cholesteryl Esters in Medium HDL	Lipoprotein subclasses
142	Concentration of Large HDL Particles	Lipoprotein subclasses
143	Free Cholesterol in Large HDL	Lipoprotein subclasses
144	Cholesteryl Esters in Large HDL	Lipoprotein subclasses
145	Phospholipids in Large HDL	Lipoprotein subclasses
146	Total Lipids in Large HDL	Lipoprotein subclasses
147	Phospholipids in Very Large HDL	Lipoprotein subclasses
148	Cholesterol in Large HDL	Lipoprotein subclasses
149	Total Lipids in Very Large HDL	Lipoprotein subclasses
150	Cholesteryl Esters in Very Large HDL	Lipoprotein subclasses
151	Cholesterol in Very Large HDL	Lipoprotein subclasses
152	Free Cholesterol in Very Large HDL	Lipoprotein subclasses
153	Phosphoglycerides	Other lipids
154	Total Cholines	Other lipids
155	Phosphatidylcholines	Other lipids
156	Sphingomyelins	Other lipids
157	Total Phospholipids in Lipoprotein Particles	Phospholipids
158	Phospholipids in VLDL	Phospholipids
159	Phospholipids in LDL	Phospholipids
160	Phospholipids in HDL	Phospholipids
161	Total Lipids in Lipoprotein Particles	Total lipids

162	Total Lipids in VLDL	Total lipids
163	Total Lipids in LDL	Total lipids
164	Total Lipids in HDL	Total lipids
165	Triglycerides in LDL	Triglycerides
166	Total Triglycerides	Triglycerides
167	Triglycerides in VLDL	Triglycerides
168	Triglycerides in HDL	Triglycerides
169	Cholesteryl esters to total lipids ratio in chylomicrons and extremely large VLDL	Lipid ratios in lipoprotein subclasses
170	Cholesteryl esters to total lipids ratio in small LDL	Lipid ratios in lipoprotein subclasses
171	Phospholipids to total lipids ratio in small LDL	Lipid ratios in lipoprotein subclasses
172	Phospholipids to total lipids ratio in medium LDL	Lipid ratios in lipoprotein subclasses
173	Cholesterol to total lipids ratio in chylomicrons and extremely large VLDL	Lipid ratios in lipoprotein subclasses
174	Triglycerides to total lipids ratio in chylomicrons and extremely large VLDL	Lipid ratios in lipoprotein subclasses
175	Cholesteryl esters to total lipids ratio in medium LDL	Lipid ratios in lipoprotein subclasses
176	Ratio of monounsaturated fatty acids to total fatty acids	Lipid ratios in lipoprotein subclasses
177	Triglycerides to total lipids ratio in very large HDL	Lipid ratios in lipoprotein subclasses
178	Ratio of polyunsaturated fatty acids to monounsaturated fatty acids	Lipid ratios in lipoprotein subclasses
179	Phospholipids to total lipids ratio in IDL	Lipid ratios in lipoprotein subclasses
180	Phospholipids to total lipids ratio in very large VLDL	Lipid ratios in lipoprotein subclasses
181	Free cholesterol to total lipids ratio in medium LDL	Lipid ratios in lipoprotein subclasses
182	Ratio of docosahexaenoic acid to total fatty acids	Lipid ratios in lipoprotein subclasses
183	Triglycerides to total lipids ratio in small HDL	Lipid ratios in lipoprotein subclasses
184	Triglycerides to total lipids ratio in large HDL	Lipid ratios in lipoprotein subclasses
185	Triglycerides to total lipids ratio in medium HDL	Lipid ratios in lipoprotein subclasses
186	Cholesteryl esters to total lipids ratio in medium HDL	Lipid ratios in lipoprotein subclasses
187	Ratio of triglycerides to phosphoglycerides	Lipid ratios in lipoprotein subclasses
188	Phospholipids to total lipids ratio in chylomicrons and extremely large VLDL	Lipid ratios in lipoprotein subclasses
189	Phospholipids to total lipids ratio in large VLDL	Lipid ratios in lipoprotein subclasses
190	Cholesteryl esters to total lipids ratio in large HDL	Lipid ratios in lipoprotein subclasses
191	Cholesteryl esters to total lipids ratio in small HDL	Lipid ratios in lipoprotein subclasses

192	Free cholesterol to total lipids ratio in small LDL	Lipid ratios in lipoprotein subclasses
193	Ratio of apolipoprotein B to apolipoprotein A1	Lipid ratios in lipoprotein subclasses
194	Phospholipids to total lipids ratio in very large HDL	Lipid ratios in lipoprotein subclasses
195	Cholesterol to total lipids ratio in large HDL	Lipid ratios in lipoprotein subclasses
196	Cholesteryl esters to total lipids ratio in very large VLDL	Lipid ratios in lipoprotein subclasses
197	Cholesterol to total lipids ratio in very large VLDL	Lipid ratios in lipoprotein subclasses
198	Free cholesterol to total lipids ratio in small HDL	Lipid ratios in lipoprotein subclasses
199	Free cholesterol to total lipids ratio in medium HDL	Lipid ratios in lipoprotein subclasses
200	Cholesterol to total lipids ratio in medium HDL	Lipid ratios in lipoprotein subclasses
201	Cholesterol to total lipids ratio in small HDL	Lipid ratios in lipoprotein subclasses
202	Triglycerides to total lipids ratio in small LDL	Lipid ratios in lipoprotein subclasses
203	Free cholesterol to total lipids ratio in chylomicrons and extremely large VLDL	Lipid ratios in lipoprotein subclasses
204	Ratio of polyunsaturated fatty acids to total fatty acids	Lipid ratios in lipoprotein subclasses
205	Phospholipids to total lipids ratio in large LDL	Lipid ratios in lipoprotein subclasses
206	Cholesterol to total lipids ratio in small LDL	Lipid ratios in lipoprotein subclasses
207	Ratio of omega-6 fatty acids to total fatty acids	Lipid ratios in lipoprotein subclasses
208	Phospholipids to total lipids ratio in very small VLDL	Lipid ratios in lipoprotein subclasses
209	Free cholesterol to total lipids ratio in very large HDL	Lipid ratios in lipoprotein subclasses
210	Triglycerides to total lipids ratio in very large VLDL	Lipid ratios in lipoprotein subclasses
211	Free cholesterol to total lipids ratio in very large VLDL	Lipid ratios in lipoprotein subclasses
212	Cholesteryl esters to total lipids ratio in very large HDL	Lipid ratios in lipoprotein subclasses
213	Free cholesterol to total lipids ratio in large LDL	Lipid ratios in lipoprotein subclasses
214	Cholesteryl esters to total lipids ratio in medium VLDL	Lipid ratios in lipoprotein subclasses
215	Phospholipids to total lipids ratio in large HDL	Lipid ratios in lipoprotein subclasses
216	Triglycerides to total lipids ratio in large LDL	Lipid ratios in lipoprotein subclasses
217	Triglycerides to total lipids ratio in medium LDL	Lipid ratios in lipoprotein subclasses
218	Triglycerides to total lipids ratio in very small VLDL	Lipid ratios in lipoprotein subclasses

219	Ratio of linoleic acid to total fatty acids	Lipid ratios in lipoprotein subclasses
220	Cholesterol to total lipids ratio in medium VLDL	Lipid ratios in lipoprotein subclasses
221	Cholesteryl esters to total lipids ratio in IDL	Lipid ratios in lipoprotein subclasses
222	Cholesteryl esters to total lipids ratio in large VLDL	Lipid ratios in lipoprotein subclasses
223	Triglycerides to total lipids ratio in large VLDL	Lipid ratios in lipoprotein subclasses
224	Phospholipids to total lipids ratio in medium HDL	Lipid ratios in lipoprotein subclasses
225	Cholesteryl esters to total lipids ratio in very small VLDL	Lipid ratios in lipoprotein subclasses
226	Cholesterol to total lipids ratio in medium LDL	Lipid ratios in lipoprotein subclasses
227	Free cholesterol to total lipids ratio in large HDL	Lipid ratios in lipoprotein subclasses
228	Cholesteryl esters to total lipids ratio in large LDL	Lipid ratios in lipoprotein subclasses
229	Phospholipids to total lipids ratio in medium VLDL	Lipid ratios in lipoprotein subclasses
230	Ratio of omega-3 fatty acids to total fatty acids	Lipid ratios in lipoprotein subclasses
231	Cholesterol to total lipids ratio in very small VLDL	Lipid ratios in lipoprotein subclasses
232	Cholesterol to total lipids ratio in IDL	Lipid ratios in lipoprotein subclasses
233	Triglycerides to total lipids ratio in IDL	Lipid ratios in lipoprotein subclasses
234	Free cholesterol to total lipids ratio in very small VLDL	Lipid ratios in lipoprotein subclasses
235	Triglycerides to total lipids ratio in medium VLDL	Lipid ratios in lipoprotein subclasses
236	Ratio of saturated fatty acids to total fatty acids	Lipid ratios in lipoprotein subclasses
237	Cholesterol to total lipids ratio in large VLDL	Lipid ratios in lipoprotein subclasses
238	Free cholesterol to total lipids ratio in IDL	Lipid ratios in lipoprotein subclasses
239	Ratio of omega-6 fatty acids to omega-3 fatty acids	Lipid ratios in lipoprotein subclasses
240	Cholesterol to total lipids ratio in large LDL	Lipid ratios in lipoprotein subclasses
241	Free cholesterol to total lipids ratio in small VLDL	Lipid ratios in lipoprotein subclasses
242	Free cholesterol to total lipids ratio in large VLDL	Lipid ratios in lipoprotein subclasses
243	Cholesterol to total lipids ratio in very large HDL	Lipid ratios in lipoprotein subclasses
244	Cholesterol to total lipids ratio in small VLDL	Lipid ratios in lipoprotein subclasses
245	Phospholipids to total lipids ratio in small VLDL	Lipid ratios in lipoprotein subclasses

246	Triglycerides to total lipids ratio in small VLDL	Lipid ratios in lipoprotein subclasses
247	Cholesteryl esters to total lipids ratio in small VLDL	Lipid ratios in lipoprotein subclasses
248	Phospholipids to total lipids ratio in small HDL	Lipid ratios in lipoprotein subclasses
249	Free cholesterol to total lipids ratio in medium VLDL	Lipid ratios in lipoprotein subclasses

Supplementary Table 2. Baseline characteristics of 109,991 participants stratified by prevalent PD at baseline.

Baseline characteristics	Total	Non-PD	PD	P value
N	109,991	109,790	201	
Age, mean(SD), years	56.5 (8.10)	56.5 (8.10)	61.9 (5.48)	**<0.001**
Gender, N(%)				**<0.001**
Female	59,141 (53.8)	59,073 (53.8)	68 (33.8)	
Male	50,850 (46.2)	50,717 (46.2)	133 (66.2)	
Smoking status, N(%)				0.110
Never	59,824 (54.7)	59,704 (54.7)	120 (60.3)	
Prior/current	49,603 (45.3)	49,524 (45.3)	79 (39.7)	
BMI, mean(SD)	27.4 (4.77)	27.4 (4.77)	26.9 (4.35)	0.092
Systolic blood pressure, mean(SD), mmHg	137.7 (18.5)	137.7 (18.5)	134.2 (17.4)	**0.009**
Currently on anti-hypertensive treatments, N(%)				0.222
No	99,555 (90.5)	99,368 (90.5)	187 (93.0)	
Yes	10,436 (9.49)	10,422 (9.49)	14 (6.97)	
History of diabetes mellitus, N(%)				0.216
No	103,327 (93.9)	103,134 (93.9)	193 (96.0)	

Yes	6,664 (6.06)	6,656 (6.06)	8 (3.98)	
History of hyperlipidemia, N(%)				0.472
No	59,455 (53.7)	59,342 (53.7)	113 (56.2)	
Yes	51,275 (46.3)	51,187 (46.3)	88 (43.8)	
Prior stroke, N(%)				**<0.001**
No	108,450 (98.6)	108,263 (98.6)	187 (93.0)	
Yes	1,541 (1.40)	1,527 (1.39)	14 (6.97)	
Use of psychotropic medications, N(%)				**<0.001**
No	101,135 (92.0)	100,974 (92.0)	161 (80.1)	
Yes	8,856 (8.05)	8,816 (8.03)	40 (19.9)	
Presence of APOE4 allelic variant(s), N(%)				0.348
No	83,342(75.8)	83,184 (75.8)	158 (78.6)	
Yes	26,649 (24.2)	26,606 24.2)	43 (21.4)	
Presence of GBA1 allelic variant(s), N(%)				0.270
No	104,707 (95.2)	104,519 (95.2)	188 (93.5)	
Yes	5,284 (4.80)	5,271 (4.80)	13 (6.47)	

Supplementary Table 3. Metabolites associated with incident PD after adjusting for all variables in main analysis and frailty index.

Metabolites	Group	HR (95% CI)	P value
Triglycerides in Very Large HDL	Lipoprotein subclasses	0.894 (0.808-0.989)	0.030
Triglycerides in Small LDL	Lipoprotein subclasses	0.883 (0.795-0.98)	0.020
Cholesteryl Esters in Small LDL	Lipoprotein subclasses	0.881 (0.797-0.973)	0.012
Cholesterol in Small LDL	Lipoprotein subclasses	0.889 (0.806-0.981)	0.019
Phospholipids in Small LDL	Lipoprotein subclasses	0.897 (0.813-0.989)	0.028
Total Lipids in Small LDL	Lipoprotein subclasses	0.886 (0.803-0.977)	0.015
Concentration of Small LDL Particles	Lipoprotein subclasses	0.886 (0.802-0.979)	0.018
Triglycerides in Medium LDL	Lipoprotein subclasses	0.874 (0.787-0.972)	0.013
Cholesteryl Esters in Medium LDL	Lipoprotein subclasses	0.883 (0.8-0.974)	0.013
Cholesterol in Medium LDL	Lipoprotein subclasses	0.889 (0.806-0.979)	0.017
Phospholipids in Medium LDL	Lipoprotein subclasses	0.891 (0.807-0.983)	0.022
Total Lipids in Medium LDL	Lipoprotein subclasses	0.886 (0.804-0.976)	0.014
Concentration of Medium LDL Particles	Lipoprotein subclasses	0.892 (0.807-0.985)	0.024
Triglycerides in Large LDL	Lipoprotein subclasses	0.881 (0.793-0.978)	0.018
Cholesteryl Esters in Large LDL	Lipoprotein subclasses	0.902 (0.818-0.994)	0.037
Cholesterol in Large LDL	Lipoprotein subclasses	0.904 (0.821-0.996)	0.041
Phospholipids in Large LDL	Lipoprotein subclasses	0.901 (0.816-0.994)	0.037
Total Lipids in Large LDL	Lipoprotein subclasses	0.898 (0.816-0.989)	0.029
Triglycerides in IDL	Lipoprotein subclasses	0.894 (0.806-0.991)	0.033
Cholesteryl Esters in IDL	Lipoprotein subclasses	0.904 (0.818-0.998)	0.046
Cholesterol in IDL	Lipoprotein subclasses	0.905 (0.82-0.999)	0.047
Total Lipids in IDL	Lipoprotein subclasses	0.898 (0.814-0.991)	0.033
Concentration of IDL Particles	Lipoprotein subclasses	0.891 (0.806-0.985)	0.024
Triglycerides in Very Small VLDL	Lipoprotein subclasses	0.901 (0.813-0.999)	0.048
Free Cholesterol in Very Small VLDL	Lipoprotein subclasses	0.902 (0.815-0.998)	0.045
Phospholipids in Very Small VLDL	Lipoprotein subclasses	0.903 (0.816-0.998)	0.046
Total Lipids in Very Small VLDL	Lipoprotein subclasses	0.899 (0.813-0.993)	0.036
Concentration of Very Small VLDL Particles	Lipoprotein subclasses	0.897 (0.81-0.992)	0.034
Triglycerides in Small VLDL	Lipoprotein subclasses	0.9 (0.813-0.997)	0.043
Free Cholesterol in Small VLDL	Lipoprotein subclasses	0.902 (0.816-0.997)	0.044
Cholesteryl Esters in Small VLDL	Lipoprotein subclasses	0.903 (0.817-0.999)	0.048

Cholesterol in Small VLDL	Lipoprotein subclasses	0.902 (0.816-0.997)	0.043
Phospholipids in Small VLDL	Lipoprotein subclasses	0.892 (0.807-0.987)	0.027
Total Lipids in Small VLDL	Lipoprotein subclasses	0.889 (0.804-0.983)	0.022
Concentration of Small VLDL Particles	Lipoprotein subclasses	0.894 (0.807-0.99)	0.031
Triglycerides in Medium VLDL	Lipoprotein subclasses	0.892 (0.806-0.987)	0.026
Free Cholesterol in Medium VLDL	Lipoprotein subclasses	0.895 (0.81-0.99)	0.030
Phospholipids in Medium VLDL	Lipoprotein subclasses	0.889 (0.805-0.982)	0.021
Total Lipids in Medium VLDL	Lipoprotein subclasses	0.886 (0.803-0.977)	0.015
Concentration of Medium VLDL Particles	Lipoprotein subclasses	0.886 (0.801-0.981)	0.019
Triglycerides in Large VLDL	Lipoprotein subclasses	0.897 (0.81-0.993)	0.035
Free Cholesterol in Large VLDL	Lipoprotein subclasses	0.889 (0.802-0.987)	0.027
Cholesteryl Esters in Large VLDL	Lipoprotein subclasses	0.897 (0.81-0.992)	0.035
Cholesterol in Large VLDL	Lipoprotein subclasses	0.892 (0.805-0.988)	0.028
Phospholipids in Large VLDL	Lipoprotein subclasses	0.894 (0.806-0.992)	0.034
Total Lipids in Large VLDL	Lipoprotein subclasses	0.893 (0.807-0.989)	0.030
Concentration of Large VLDL Particles	Lipoprotein subclasses	0.889 (0.801-0.986)	0.027
Triglycerides in Very Large VLDL	Lipoprotein subclasses	0.901 (0.813-0.999)	0.049
Free Cholesterol in Very Large VLDL	Lipoprotein subclasses	0.893 (0.805-0.99)	0.032
Cholesteryl Esters in Very Large VLDL	Lipoprotein subclasses	0.897 (0.809-0.994)	0.038
Cholesterol in Very Large VLDL	Lipoprotein subclasses	0.893 (0.806-0.99)	0.031
Phospholipids in Very Large VLDL	Lipoprotein subclasses	0.894 (0.806-0.992)	0.035
Total Lipids in Very Large VLDL	Lipoprotein subclasses	0.897 (0.809-0.994)	0.038
Concentration of Very Large VLDL Particles	Lipoprotein subclasses	0.893 (0.804-0.991)	0.034
Glycoprotein Acetyls	Inflammation	0.875 (0.791-0.968)	0.009
Glutamine	Amino acids	1.122 (1.023-1.23)	0.015
Linoleic Acid	Fatty acids	0.859 (0.781-0.944)	0.002
Saturated Fatty Acids	Fatty acids	0.881 (0.799-0.971)	0.011
Monounsaturated Fatty Acids	Fatty acids	0.866 (0.784-0.956)	0.004
Polyunsaturated Fatty Acids	Fatty acids	0.861 (0.781-0.95)	0.003
Omega-6 Fatty Acids	Fatty acids	0.854 (0.775-0.942)	0.002
Total Fatty Acids	Fatty acids	0.869 (0.791-0.954)	0.003
Apolipoprotein B	Apolipoproteins	0.893 (0.81-0.984)	0.022
Phosphatidylcholines	Other lipids	0.899 (0.812-0.995)	0.040
Total Cholines	Other lipids	0.894 (0.808-0.989)	0.029

Phosphoglycerides	Other lipids	0.893 (0.808-0.987)	0.027
Concentration of LDL Particles	Lipoprotein particle concentrations	0.899 (0.813-0.993)	0.036
Concentration of VLDL Particles	Lipoprotein particle concentrations	0.884 (0.799-0.979)	0.018
Total Lipids in LDL	Total lipids	0.893 (0.812-0.982)	0.019
Total Lipids in VLDL	Total lipids	0.887 (0.804-0.979)	0.017
Total Lipids in Lipoprotein Particles	Total lipids	0.88 (0.801-0.967)	0.008
Free Cholesterol in VLDL	Free cholesterol	0.884 (0.8-0.978)	0.016
Total Free Cholesterol	Free cholesterol	0.888 (0.805-0.98)	0.018
Cholesteryl Esters in LDL	Cholesteryl esters	0.893 (0.812-0.983)	0.021
Cholesteryl Esters in VLDL	Cholesteryl esters	0.896 (0.812-0.989)	0.029
Total Esterified Cholesterol	Cholesteryl esters	0.899 (0.815-0.991)	0.032
Phospholipids in LDL	Phospholipids	0.896 (0.813-0.987)	0.027
Phospholipids in VLDL	Phospholipids	0.886 (0.801-0.979)	0.017
Total Phospholipids in Lipoprotein Particles	Phospholipids	0.888 (0.804-0.981)	0.020
Triglycerides in LDL	Triglycerides	0.878 (0.791-0.975)	0.015
Triglycerides in VLDL	Triglycerides	0.899 (0.813-0.994)	0.038
Total Triglycerides	Triglycerides	0.893 (0.808-0.988)	0.028
LDL Cholesterol	Cholesterol	0.898 (0.817-0.987)	0.026
Clinical LDL Cholesterol	Cholesterol	0.906 (0.825-0.995)	0.039
VLDL Cholesterol	Cholesterol	0.889 (0.806-0.981)	0.019
Remnant Cholesterol (Non-HDL, Non-LDL -Cholesterol)	Cholesterol	0.888 (0.806-0.977)	0.015
Total Cholesterol Minus HDL-C	Cholesterol	0.891 (0.81-0.979)	0.017
Total Cholesterol	Cholesterol	0.896 (0.813-0.986)	0.025
Cholesteryl esters to total lipids ratio in small LDL	—	0.923 (0.854-0.997)	0.041
Triglycerides to total lipids ratio in chylomicrons and extremely large VLDL	—	0.91 (0.837-0.99)	0.027
Cholesteryl esters to total lipids ratio in chylomicrons and extremely large VLDL	—	1.12 (1.029-1.219)	0.009
Cholesterol to total lipids ratio in chylomicrons and extremely large VLDL	—	1.102 (1.011-1.202)	0.028

Supplementary Table 4. Overlapping and distinct metabolites associated with incident and prevalent PD.

**Metabolites**	**Group**	**HR (95%CI)**	**P value**	**OR (95% CI)**	**P value**
**Overlap**		**Incident PD**		**Prevalent PD**	
Polyunsaturated Fatty Acids	Fatty acids	0.864 (0.793-0.942)	**8.98×10^{-4}**	0.723 (0.619-0.845)	**4.47×10^{-5}**
Omega-6 Fatty Acids	Fatty acids	0.865 (0.794-0.942)	**8.71×10^{-4}**	0.78 (0.668-0.911)	0.002
Total Fatty Acids	Fatty acids	0.878 (0.808-0.955)	0.002	0.757 (0.652-0.88)	**2.72×10^{-4}**
Free Cholesterol in Small HDL	Lipoprotein subclasses	0.882 (0.807-0.964)	0.005	0.739 (0.627-0.871)	**3.20×10^{-4}**
Total Lipids in Small HDL	Lipoprotein subclasses	0.891 (0.818-0.971)	0.008	0.744 (0.638-0.869)	**1.84×10^{-4}**
Concentration of Small HDL Particles	Lipoprotein subclasses	0.893 (0.819-0.974)	0.010	0.753 (0.642-0.882)	**4.59×10^{-4}**
Phospholipids in Small HDL	Lipoprotein subclasses	0.895 (0.821-0.975)	0.012	0.754 (0.643-0.884)	**5.05×10^{-4}**
Total Cholesterol	Cholesterol	0.905 (0.832-0.986)	0.022	0.827 (0.709-0.964)	0.015
LDL Cholesterol	Cholesterol	0.907 (0.835-0.987)	0.023	0.813 (0.699-0.946)	0.007
Total Cholesterol Minus HDL-C	Cholesterol	0.911 (0.838-0.99)	0.027	0.811 (0.698-0.942)	0.006
Remnant Cholesterol (Non-HDL, Non-LDL -Cholesterol)	Cholesterol	0.918 (0.844-0.999)	0.047	0.819 (0.702-0.956)	0.011
Clinical LDL Cholesterol	Cholesterol	0.92 (0.847-0.999)	0.048	0.834 (0.719-0.968)	0.017
Cholesteryl Esters in LDL	Cholesteryl esters	0.903 (0.83-0.982)	0.017	0.803 (0.689-0.935)	0.005
Total Esterified Cholesterol	Cholesteryl esters	0.906 (0.831-0.987)	0.023	0.829 (0.71-0.968)	0.018
Linoleic Acid	Fatty acids	0.874 (0.804-0.949)	0.001	0.83 (0.714-0.965)	0.015
Monounsaturated Fatty Acids	Fatty acids	0.889 (0.815-0.969)	0.008	0.775 (0.658-0.913)	0.002
Saturated Fatty Acids	Fatty acids	0.891 (0.818-0.97)	0.008	0.793 (0.676-0.931)	0.005
Total Free Cholesterol	Free cholesterol	0.906 (0.831-0.988)	0.025	0.826 (0.705-0.966)	0.017
Free Cholesterol in VLDL	Free cholesterol	0.915 (0.839-0.998)	0.046	0.823 (0.699-0.969)	0.019
Total Concentration of Lipoprotein Particles	Lipoprotein particle concentrations	0.896 (0.817-0.982)	0.019	0.826 (0.697-0.979)	0.027
Cholesteryl Esters in Medium LDL	Lipoprotein subclasses	0.897 (0.823-0.978)	0.013	0.794 (0.677-0.93)	0.004
Total Lipids in Medium LDL	Lipoprotein subclasses	0.899 (0.825-0.979)	0.014	0.787 (0.674-0.92)	0.003

Cholesterol in Medium LDL	Lipoprotein subclasses	0.901 (0.827-0.981)	0.016	0.799 (0.684-0.935)	0.005
Cholesterol in Small HDL	Lipoprotein subclasses	0.902 (0.828-0.982)	0.017	0.771 (0.661-0.899)	0.001
Cholesteryl Esters in Small LDL	Lipoprotein subclasses	0.9 (0.825-0.982)	0.018	0.812 (0.691-0.955)	0.012
Triglycerides in Medium VLDL	Lipoprotein subclasses	0.9 (0.824-0.983)	0.019	0.823 (0.697-0.972)	0.022
Phospholipids in Medium LDL	Lipoprotein subclasses	0.903 (0.828-0.985)	0.021	0.785 (0.669-0.922)	0.003
Total Lipids in Medium VLDL	Lipoprotein subclasses	0.905 (0.831-0.986)	0.022	0.811 (0.692-0.951)	0.010
Total Lipids in Small LDL	Lipoprotein subclasses	0.906 (0.832-0.987)	0.024	0.821 (0.7-0.963)	0.015
Triglycerides in Medium LDL	Lipoprotein subclasses	0.902 (0.824-0.987)	0.025	0.761 (0.634-0.915)	0.004
Cholesteryl Esters in Large LDL	Lipoprotein subclasses	0.907 (0.833-0.988)	0.026	0.811 (0.694-0.948)	0.008
Cholesterol in Small LDL	Lipoprotein subclasses	0.907 (0.832-0.988)	0.026	0.824 (0.703-0.967)	0.018
Total Lipids in Large LDL	Lipoprotein subclasses	0.909 (0.835-0.989)	0.027	0.806 (0.691-0.939)	0.006
Total Lipids in Large VLDL	Lipoprotein subclasses	0.906 (0.829-0.989)	0.028	0.846 (0.716-0.999)	0.049
Triglycerides in Small LDL	Lipoprotein subclasses	0.906 (0.828-0.991)	0.031	0.828 (0.693-0.988)	0.037
Cholesterol in Large LDL	Lipoprotein subclasses	0.911 (0.837-0.992)	0.031	0.82 (0.702-0.957)	0.012
Cholesteryl Esters in Small HDL	Lipoprotein subclasses	0.913 (0.839-0.993)	0.034	0.791 (0.68-0.921)	0.002
Phospholipids in Large LDL	Lipoprotein subclasses	0.915 (0.839-0.998)	0.044	0.811 (0.692-0.951)	0.010
Concentration of Medium VLDL Particles	Lipoprotein subclasses	0.914 (0.837-0.998)	0.044	0.827 (0.701-0.976)	0.025
Free Cholesterol in Medium LDL	Lipoprotein subclasses	0.916 (0.841-0.998)	0.046	0.823 (0.703-0.965)	0.016
Concentration of Medium LDL Particles	Lipoprotein subclasses	0.915 (0.839-0.999)	0.046	0.842 (0.716-0.991)	0.038
Phosphoglycerides	Other lipids	0.884 (0.809-0.966)	0.006	0.791 (0.673-0.929)	0.004
Phosphatidylcholines	Other lipids	0.885 (0.81-0.968)	0.007	0.812 (0.691-0.956)	0.012
Total Cholines	Other lipids	0.886 (0.811-0.969)	0.008	0.804 (0.684-0.946)	0.008
Total Phospholipids in Lipoprotein Particles	Phospholipids	0.889 (0.815-0.971)	0.009	0.803 (0.685-0.942)	0.007
Phospholipids in LDL	Phospholipids	0.911 (0.836-0.992)	0.032	0.805 (0.689-0.942)	0.007
Phospholipids in VLDL	Phospholipids	0.914 (0.838-0.997)	0.044	0.811 (0.689-0.955)	0.012

Total Lipids in Lipoprotein Particles	Total lipids	0.889 (0.819-0.966)	0.005	0.796 (0.687-0.923)	0.003
Total Lipids in LDL	Total lipids	0.905 (0.833-0.984)	0.020	0.8 (0.689-0.93)	0.004
Total Lipids in VLDL	Total lipids	0.907 (0.832-0.988)	0.026	0.804 (0.686-0.943)	0.007
Total Triglycerides	Triglycerides	0.907 (0.831-0.99)	0.029	0.806 (0.682-0.951)	0.011
Triglycerides in VLDL	Triglycerides	0.909 (0.832-0.992)	0.033	0.837 (0.709-0.988)	0.035
Triglycerides in LDL	Triglycerides	0.91 (0.832-0.996)	0.040	0.742 (0.619-0.89)	0.001
Phospholipids to total lipids ratio in small LDL	—	1.095 (1.014-1.183)	0.021	1.203 (1.071-1.35)	0.002
Phospholipids to total lipids ratio in IDL	—	1.095 (1.011-1.185)	0.026	1.158 (1.002-1.338)	0.047
Cholesteryl esters to total lipids ratio in small LDL	—	0.923 (0.86-0.991)	0.027	0.86 (0.782-0.944)	0.002
**Incident PD only**					
Concentration of HDL Particles	Lipoprotein particle concentrations	0.905 (0.826-0.992)	0.033		
Triglycerides in Large VLDL	Lipoprotein subclasses	0.901 (0.825-0.984)	0.021		
Concentration of Large VLDL Particles	Lipoprotein subclasses	0.905 (0.827-0.99)	0.030		
Triglycerides in Very Large VLDL	Lipoprotein subclasses	0.909 (0.831-0.994)	0.037		
Concentration of Very Large VLDL Particles	Lipoprotein subclasses	0.908 (0.829-0.994)	0.037		
Free Cholesterol in Large VLDL	Lipoprotein subclasses	0.909 (0.831-0.995)	0.038		
Total Lipids in Very Large VLDL	Lipoprotein subclasses	0.91 (0.833-0.995)	0.039		
Concentration of Small LDL Particles	Lipoprotein subclasses	0.913 (0.837-0.996)	0.040		
Phospholipids in Large VLDL	Lipoprotein subclasses	0.912 (0.834-0.998)	0.044		
Phospholipids in Very Large VLDL	Lipoprotein subclasses	0.914 (0.835-1)	0.050		
Cholesteryl esters to total lipids ratio in chylomicrons and extremely large VLDL	—	1.086 (1.005-1.174)	0.036		

Free cholesterol to total lipids ratio in very large VLDL	—	1.074 (1.001-1.152)	0.047		
**Prevalent PD only**					
Omega-3 Fatty Acids	Fatty acids			0.669 (0.565-0.791)	**2.76×10^{-6}**
Docosahexaenoic Acid	Fatty acids			0.676 (0.568-0.803)	**8.77×10^{-6}**
Phospholipids to total lipids ratio in large VLDL	—			0.791 (0.705-0.887)	**6.25×10^{-5}**
Ratio of omega-6 fatty acids to omega-3 fatty acids	—			1.31 (1.142-1.503)	**1.19×10^{-4}**
Ratio of omega-3 fatty acids to total fatty acids	—			0.729 (0.616-0.861)	**2.04×10^{-4}**
Triglycerides to total lipids ratio in large VLDL	—			1.261 (1.106-1.438)	**5.22×10^{-4}**
Valine	Amino acids			0.743 (0.628-0.879)	**5.51×10^{-4}**
Tyrosine	Amino acids			0.755 (0.642-0.888)	**6.95×10^{-4}**
Triglycerides in Large LDL	Lipoprotein subclasses			0.731 (0.61-0.876)	**7.11×10^{-4}**
Phospholipids to total lipids ratio in chylomicrons and extremely large VLDL	—			0.798 (0.698-0.913)	0.001
Free cholesterol to total lipids ratio in large VLDL	—			0.804 (0.706-0.916)	0.001
Total Concentration of Branched-Chain Amino Acids (Leucine + Isoleucine + Valine)	Amino acids			0.754 (0.637-0.893)	0.001
Total Lipids in Small VLDL	Lipoprotein subclasses			0.766 (0.651-0.903)	0.001
Triglycerides in IDL	Lipoprotein subclasses			0.749 (0.627-0.895)	0.001
Leucine	Amino acids			0.762 (0.643-0.903)	0.002
Creatinine	Fluid balance			0.759 (0.632-0.911)	0.003
Triglycerides in Very Small VLDL	Lipoprotein subclasses			0.768 (0.644-0.916)	0.003
Triglycerides in Small HDL	Lipoprotein subclasses			0.77 (0.646-0.918)	0.004
Phospholipids in Small VLDL	Lipoprotein subclasses			0.788 (0.668-0.929)	0.005

Concentration of Small VLDL Particles	Lipoprotein subclasses	0.786 (0.663-0.931)	0.005
Triglycerides in Small VLDL	Lipoprotein subclasses	0.784 (0.661-0.931)	0.005
Glycoprotein Acetyls	Inflammation	0.795 (0.676-0.935)	0.006
Cholesteryl Esters in Small VLDL	Lipoprotein subclasses	0.798 (0.677-0.94)	0.007
Cholesterol in Small VLDL	Lipoprotein subclasses	0.8 (0.68-0.942)	0.007
Phenylalanine	Amino acids	0.806 (0.686-0.947)	0.009
Triglycerides in HDL	Triglycerides	0.799 (0.675-0.946)	0.009
Triglycerides in Medium HDL	Lipoprotein subclasses	0.8 (0.676-0.948)	0.010
Phospholipids to total lipids ratio in very large HDL	—	1.318 (1.066-1.63)	0.011
Ratio of docosahexaenoic acid to total fatty acids	—	0.81 (0.69-0.953)	0.011
Free Cholesterol in Small VLDL	Lipoprotein subclasses	0.812 (0.69-0.957)	0.013
VLDL Cholesterol	Cholesterol	0.819 (0.699-0.959)	0.013
Total Lipids in Very Small VLDL	Lipoprotein subclasses	0.814 (0.692-0.958)	0.014
Concentration of VLDL Particles	Lipoprotein particle concentrations	0.811 (0.686-0.958)	0.014
Phospholipids to total lipids ratio in large HDL	—	0.843 (0.736-0.966)	0.014
Ratio of omega-6 fatty acids to total fatty acids	—	1.089 (1.017-1.165)	0.014
Phospholipids to total lipids ratio in very large VLDL	—	0.862 (0.766-0.971)	0.015
Glutamine	Amino acids	1.2 (1.036-1.39)	0.015
Average Diameter for HDL Particles	Lipoprotein particle si—es	1.358 (1.061-1.738)	0.015
Free cholesterol to total lipids ratio in medium LDL	—	1.232 (1.041-1.458)	0.015
Degree of Unsaturation	Fatty acids	0.831 (0.715-0.966)	0.016
Apolipoprotein B	Apolipoproteins	0.827 (0.706-0.967)	0.018

Triglycerides to total lipids ratio in very large HDL	—	0.777 (0.63-0.957)	0.018
Phospholipids in Medium VLDL	Lipoprotein subclasses	0.823 (0.7-0.968)	0.019
Concentration of Very Small VLDL Particles	Lipoprotein subclasses	0.822 (0.696-0.97)	0.020
Cholesteryl Esters in VLDL	Cholesteryl esters	0.829 (0.707-0.972)	0.021
Total Lipids in IDL	Lipoprotein subclasses	0.83 (0.708-0.973)	0.021
Phospholipids in Very Large HDL	Lipoprotein subclasses	1.203 (1.025-1.412)	0.024
Phospholipids in Very Small VLDL	Lipoprotein subclasses	0.827 (0.702-0.976)	0.024
Concentration of Large LDL Particles	Lipoprotein subclasses	0.832 (0.706-0.98)	0.028
Triglycerides to total lipids ratio in very small VLDL	—	0.829 (0.701-0.98)	0.028
Free Cholesterol in Very Large HDL	Lipoprotein subclasses	1.187 (1.019-1.384)	0.028
Triglycerides to total lipids ratio in small HDL	—	0.831 (0.704-0.982)	0.030
Concentration of LDL Particles	Lipoprotein particle concentrations	0.835 (0.709-0.983)	0.030
Cholesteryl Esters in Large VLDL	Lipoprotein subclasses	0.833 (0.706-0.983)	0.030
Total Lipids in Very Large HDL	Lipoprotein subclasses	1.194 (1.016-1.404)	0.032
Isoleucine	Amino acids	0.836 (0.709-0.985)	0.032
Free Cholesterol in Medium VLDL	Lipoprotein subclasses	0.837 (0.712-0.985)	0.032
Cholesterol in Large VLDL	Lipoprotein subclasses	0.834 (0.706-0.986)	0.033
Free Cholesterol in LDL	Free cholesterol	0.843 (0.721-0.987)	0.033
Triglycerides to total lipids ratio in chylomicrons and extremely large VLDL	—	1.225 (1.014-1.48)	0.035
Cholesterol in Very Large HDL	Lipoprotein subclasses	1.188 (1.011-1.397)	0.036
Cholesteryl Esters in IDL	Lipoprotein subclasses	0.843 (0.718-0.989)	0.036
Phospholipids in Small LDL	Lipoprotein subclasses	0.844 (0.721-0.99)	0.037

Free Cholesterol in Very Small VLDL	Lipoprotein subclasses	0.839 (0.712-0.99)	0.037
Sphingomyelins	Other lipids	0.837 (0.708-0.99)	0.038
Lactate	Glycolysis related metabolites	0.857 (0.74-0.992)	0.038
Free Cholesterol in Large LDL	Lipoprotein subclasses	0.845 (0.72-0.992)	0.040
Cholesteryl Esters in Very Large HDL	Lipoprotein subclasses	1.184 (1.006-1.394)	0.042
Phospholipids in IDL	Lipoprotein subclasses	0.844 (0.716-0.994)	0.042
Triglycerides to total lipids ratio in medium HDL	—	0.846 (0.718-0.995)	0.044
Cholesterol in IDL	Lipoprotein subclasses	0.849 (0.724-0.996)	0.045
Free cholesterol to total lipids ratio in large LDL	—	1.194 (1.004-1.42)	0.045
Cholesteryl Esters in Very Large VLDL	Lipoprotein subclasses	0.844 (0.713-0.997)	0.046
Concentration of IDL Particles	Lipoprotein subclasses	0.847 (0.72-0.998)	0.047
Cholesterol to total lipids ratio in large HDL	—	1.198 (1.002-1.433)	0.047
Cholesteryl esters to total lipids ratio in large HDL	—	1.195 (1.002-1.425)	0.048
Free cholesterol to total lipids ratio in chylomicrons and extremely large VLDL	—	0.82 (0.673-0.999)	0.049

*Bolded P values suggested the metabolites were significantly associated with incident and/or prevalent PD after multiple testing correction.

Supplementary Figure 1. Predictive values of conventional PD risk factors, the selected metabolites, and the combined model of risk factors and selected metabolites for prediction of 10-year PD risk.

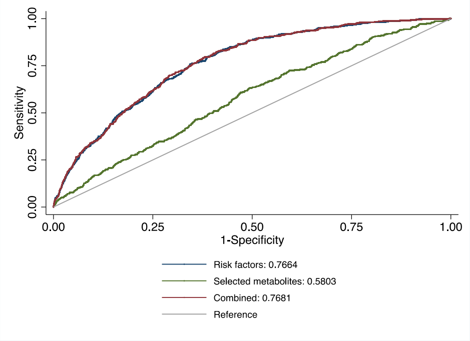

Receiver operator characteristic (ROC) curves of conventional risk factor-based model, metabolite-based model and the combined model of risk factors and metabolites were blue, green and red respectively. The AUC for conventional risk factors-based model, metabolite-based model and the combined model were 0.766 (95% CI: 0.746-0.787), 0.580 (95%CI: 0.554-0.607), and 0.768 (95% CI: 0.748-0.788). There was no significant increase in the predictive value compared to the risk-factor-based model(P=0.145).
